# Palliative Treatment in Advanced Pulmonary Tuberculosis

**Published:** 1910-01-01

**Authors:** H. Hyslop Thomson

**Affiliations:** Medical Superintendent, Liverpool Sanatorium


### Hospital Clinics.

### PALLIATIVE TREATMENT IN ADVANCED PULMONARY TUBERCULOSIS.

By H. Hyslop Thomson, M.D.; Medical Superintendent, Liverpool Sanatorium.

The distressing and painful symptoms met with in acute and advanced tuberculosis of the lungs fall into two groups. In one distinctive group pulmonary symptoms predominate, and the patient dies asphyxiated, in consequence of the gross structural changes produced by the morbid process in the lungs. In a second group toxic symptoms are chiefly in evidence, and the patient sinks overwhelmed by the intensity of the intoxication or gradually succumbs to general exhaustion and cardiac asthenia. Not infrequently, however, advanced cases are met with in which symptoms representative of both groups are present; these are usually cases of chronic phthisis with frequent acute or subacute exacerbations. The great majority of advanced consumptives are beyond the aid of curative treatment. All that remains to be done is to prolong life and ameliorate the distressing symptoms which characterise the closing stages of the disease. At the same time the importance of prophylactic measures throughout the whole course of the illness must not be overlooked, for it is in the expectoration of the advanced and incurable consumptive that we have the *fons et origo mali.*

### General Measures.

For humanitarian as well as prophylactic reasons the isolation of advanced cases of pulmonary tuberculosis amongst the poor is to be strongly recommended. When treated at home, every effort should be made to promote the patient's physical comfort and to avoid worry and mental anxiety. When circumstances permit, he should be isolated in a bright, well-ventilated room with open windows. As far as possible an equable temperature should be secured in the room, as extremes of heat and cold induce coughing, and are discomforting to the patient. A single bed with a spring mattress is to be preferred, as it is more comfortable and facilitates the many little acts of attention so necessary to the bed-ridden patient. A water-bed contributes to the comfort of the patient who is greatly emaciated and threatened with pressure sores. In advanced pulmonary tuberculosis there not infrequently takes place a marked change in the whole conduct and disposition of the patient. Irritability and morbid suspicions are perhaps the most constant features met with. When signs of mental disturbance make their appearance the patient should be cared for by a well-trained and reliable nurse.

### Diet in Advanced Consumption.

The dieting of patients with advanced pulmonary tuberculosis demands careful attention. Nothing is gained by indiscriminate feeding on a large scale; the tastes and requirements of the patient, as well as his digestive capacity, have to be carefully considered. Food is usually most readily taken when given in small quantities at frequent intervals. It is almost unnecessary to add that the cooking and method of serving have an important bearing on the likes and dislikes of the patient.

It is not desirable to outline any particular dietary for the advanced and incurable consumptive, as this must vary within considerable limits in different patients. The end to be kept in view is to sustain the strength of the patient without causing additional discomfort by overtaxing the impaired digestive and assimilative functions. If the digestive function is relatively sound, a diet approximating to that in vogue in the treatment of early sanatorium cases may be given. In patients with a poor digestive capacity light soups, milk, fish, chicken, game, tripe, pigeon, rabbit, milk pudding, junket, cooked fruit, etc., constitute a diet which is usually well tolerated. Not infrequently an intolerance to milk, butter, and other fat foods is observed, in which case they should for a time at least be reduced in quantity, or altogether eliminated from the diet. Uncooked food, consisting of raw meat, beef-juice, raw eggs, and oysters, are well borne in some cases. In severe hæmoptysis a dry restricted diet, including gelatine moulds, should be given, and in pneumothorax and advanced laryngeal disease liquid nourishment is called for.

### Distressing Localising Symptoms.

The localising symptoms which give rise to most distress in the advanced stages of pulmonary tuberculosis and call most urgently for palliative treatment are cough and dyspnœa. Severe and exhausting paroxysms of coughing, unaccompanied by the expulsion of much expectoration, are not infrequent, and call for immediate relief. For this type of cough sedatives such as morphine, codeine, or heroin, are indicated. A draught containing morphine and belladonna with a tablespoonful of old brandy administered at bedtime invariably gives marked relief and secures a good night's rest.

In cases characterised by muscular asthenia repeated short explosive efforts are necessary before the expectoration can be got rid of owing to the failing expulsive powers. If associated with this significant type of cough there are signs of commencing somnolence and evidence of failing heart the patient should be left alone. If, however, the heart is still possessed of some reserve strength and the patient suffers from the repeated and distressing efforts to get rid of the expectoration, relief should be given by the cautious administration of sedatives combined with stimulants.

In the severe attacks of coughing so frequently induced by the taking of food 15 to 20 minims of tr. belladonnæ should be given before food; at the same time the pharynx may be sprayed with a 2-per-cent. solution of cocaine. For the troublesome cough accompanied by a large amount of expectoration met with in advanced cases of mixed infection the best remedy is a mixture containing elixir calcii chloridi, guaiacol, and liq. morph. hydrochlor. The position of the patient relative to the situation of an actively secreting cavity should also be attended to as thereby considerable relief may be obtained for several hours.

Dyspnœa is a frequent and distressing symptom, especially marked in those cases of chronic type with extensive pulmonary involvement, but relatively little associated toxæmia. Fortunately the onset of severe dyspnœa during the closing phases of the disease is frequently accompanied by failing consciousness. Palliative treatment is, therefore, only called for when dyspnœa is subjectively a cause of distress. If the distress is marked, inhalations of oxygen have, in the writer's experience given marked relief; but it is only during the brief struggle prior to the onset of cardiac failure that the administration of oxygen is recommended. Should oxygen fail to give appreciable relief, and indications point to a long and painful struggle, the administration of small doses of morphine is called for; this should never be withheld for the relief of suffering in the terminal stages of hopeless cases. Tracheotomy is sometimes necessary as a measure of relief in advanced laryngeal tuberculosis. In two cases under the writer's observation it was the means of immediate and grateful relief to the patient.

### Cardiac Distress.

Symptoms of failing heart sooner or later make their appearance in all cases of advanced pulmonary tuberculosis. It arises from the effects of toxæmia and from the mechanical interference to the circulation occasioned by pulmonary fibrosis and areas of disease. Failure of the right heart is liable to occur in all chronic cases characterised by dyspnœa, lividity, and quickened pulse rate. It is usually gradual in its onset, but occasionally it develops suddenly. When consciousness is impaired no active treatment is called for. If, however, the patient is conscious, breathless, with hammering heart and throbbing veins, inhalations of oxygen should be given, but if the case is obviously hopeless no effort should be made to stimulate the flagging heart.

Sudden syncopal attacks occasionally develop which call for the administration of strychnine, ether, brandy, etc. Sudden failure of the heart is most frequently observed in cases of pulmonary tuberculosis associated with bronchial asthma. When the tuberculous mischief is not too far advanced the attack may be recovered from, as the heart responds well to inhalations of amyl nitrite and injections of ether. Most frequently, however, in advanced pulmonary tuberculosis the clinical picture is one of gradual cardiac failure, death supervening from general exhaustion and cardiac asthenia. This condition does not *per se* give rise to much distress, and calls for no active treatment.

Diarrhœa is frequently a distressing symptom. It may be due to tuberculous or amyloid disease or arise from the toxæmic condition. It is usually associated with some degree of abdominal pain. In severe cases a mixture containing opium should be given, and the diet should be modified to meet the requirements of the individual. Tuberculosis of the peritoneum may develop and give rise to a large effusion, which, by impeding the respiratory act, is the cause of much distress. Paracentesis, however, will give prompt relief.

A localised tuberculosis in the region of the cæcum or appendix is occasionally met with and is the cause of severe abdominal pain. As operative interference is contra-indicated relief must be obtained by the free use of morphine.

### Pain and Sleeplessness.

Pain, whatever its cause, must always receive appropriate treatment. Pain due to involvement of the pleura is frequent, and although not usually of an acute character is a source of much annoyance. It is best treated by mild counter-irritation and small doses of morphine. The development of herpes along the course of an inter-costal nerve is sometimes the cause of acute suffering. In such cases morphine should be administered and the affected side should be painted with some anodyne liniment.

Headache and neuralgic pains, chiefly of toxic origin, are a frequent source of discomfort, and should be met with the usual remedial measures. Pain on swallowing may give rise to much distress in cases of advanced laryngeal disease. Considerable relief may be obtained by spraying the pharynx with a weak solution of cocaine and morphine before nourishment is taken. In severe cases it is sometimes necessary to use the nasal tube for feeding purposes.

The procuring of sleep at night is an important part of the treatment of advanced consumptives. Sleepless nights are followed by exhaustion, and greatly intensify the misery of the patient. A careful preparation should be made for the night's rest. By paying strict attention to the personal comfort of the patient, the posture assumed in bed, the temperature, ventilation, and quietness of the room, much may be done to pave the way for a restful night. Sponging of the body surface should never be omitted. When the patient is unable to sleep an effort should be made to find out the cause upon which the sleeplessness depends. If it is due to pain morphine should be given in suitable doses. In asthenic cases it is usually due to exhaustion, in which case a glass of hot milk with half an ounce of brandy or a stimulating draught should be given. If due to dyspnœa the patient should be propped up in bed in the position which gives him the greatest degree of comfort, while a warm stimulating draught should also be administered.

If hypnotics are called for, veronal, combined with two grains of caffeine, might be given; in the writer's experience it has given excellent results.

### Some Terminal Complications.

Of terminal complications one of the most painful and distressing is pneumothorax. It is met with in acute and progressive cases with an active subpleural focus, but is by no means a frequent complication. The onset of pneumothorax usually gives rise to considerable shock, which is best treated by hypodermic injections of morphine and strychnine. If there is much respiratory embarrassment with cardiac displacement, paracentesis, simultaneously with the administration of oxygen, should be performed. This treatment gives great relief to the patient.

A very rare terminal complication in acute progressive cases is subcutaneous emphysema. In one case under the writer's observation it was the cause of much distress and called for the free administration of morphine. In chronic cases characterised by much pulmonary fibrosis œdema of the limbs, trunk, or face is not infrequently met with in the closing stages of the disease. In such cases the only treatment necessary is that which aims at the relief of suffering. Very exceptionally in cases of pneumothorax œdema of the arm is observed on the same side as the rupture. Tuberculous ulceration of the mouth or pharynx is sometimes the cause of great suffering, especially when food is taken. In such cases nourishment should be given in liquid form, and some relief may be obtained by spraying with a weak solution of cocaine and boric acid; but to patients in this pitiful condition the end usually comes as a welcome relief.

While the great majority of advanced and apparently incurable cases of consumption die, it occasionally happens that an apparently hopeless case recovers and is restored to some measure of health, for a time at least. Instances are known to the writer of advanced consumptives being admitted to a home for incurables and being discharged with the disease arrested and quiescent. The knowledge of this fact should hearten us to persevere with palliative treatment for the relief of suffering, and encourage us to leave no measure untried which aims at arresting the progress of the disease.

